# Thermal Responses to Exercise in Male Master Track & Field Athletes: Monitoring during Competition

**DOI:** 10.5114/jhk/218447

**Published:** 2026-04-02

**Authors:** Jakub Grzegorz Adamczyk, Bartłomiej Michalak, Łukasz Gutkowski, Jakub Bałdyka, Manuel Sillero-Quintana, Dariusz Boguszewski, Karol Gryko, Kazimierz Mikołajec, Anna Kopiczko

**Affiliations:** 1 Department of Theory of Sport, Józef Piłsudski University of Physical Education in Warsaw, Warsaw, Poland.; 2 Faculty of Physical Activity and Sports Sciences, Universidad Politecnica de Madrid, Madrid, Spain.; 3 Department of Individual Sports, Józef Piłsudski University of Physical Education in Warsaw, Warsaw, Poland.; 4 Department of Team Sport Games, Józef Piłsudski University of Physical Education in Warsaw, Warsaw, Poland.; 5 Department of Basketball and Football, Jerzy Kukuczka Academy of Physical Education in Katowice, Katowice, Poland.; 6 Department of Human Biology, Józef Piłsudski University of Physical Education in Warsaw, Warsaw, Poland.

**Keywords:** aging, thermal imaging, thermoregulation, monitoring, athletes

## Abstract

Due to involutional changes, the ability to perform physical exercise may undergo dynamic alterations. Therefore, analysis of physiological variables characterizing the response to competitive effort in Master athletes can provide additional information about the condition of the athlete and any potential dysfunctions. The aim of the study was to assess the impact of physical effort (warm-up and competition) on changes in skin surface temperature (T_sk_) of the lower limbs in athletes across different age categories. Considering the abrupt changes in the functioning of various systems, athletes were divided into three age categories: 35–45 years, 50–65 years, and over 70 years of age. Thermographic imaging was applied at rest and immediately after the race. In the 35–45 age group, a statistically significant decrease in T_sk_ was observed after exercise, particularly in the area of the rectus femoris muscle of both lower limbs, with the largest reduction recorded for the right rectus femoris muscle (ΔT_sk_ = 0.63°C). Significant changes in Tsk were also found in the left biceps femoris muscle and the right gastrocnemius muscle. In the 50–65 and 70+ age groups, the changes were not statistically significant. Overall, exercise-induced changes in lower-limb skin temperature were evident primarily in the youngest Master athletes, whereas no consistent or statistically significant age-related differences were observed among groups. These findings suggest a relative preservation of thermoregulatory responses with advancing age in trained individuals, however, given the predominantly non-significant inter-group comparisons, age-related interpretations should be considered exploratory.

## Introduction

During human development, the function of multiple physiological systems undergoes gradual yet significant changes, reflecting the dynamic nature of biological maturation and aging. Between the ages of 25 and 30, people reach their peak functional and adaptive capacity (Allen and Hopkins, 2015). After this period, a decline in the efficiency of various physiological processes is observed, including the thermoregulatory mechanism (Kenney, 2003), as well as an increase in stiffness and a simultaneous decrease in the elasticity of muscle fibers (Agyapong-Badu et al., 2016; Marcucci and Reggiani, 2020) and potential asymmetries (Marzano-Felisatti et al., 2023). Aging is also associated with deteriorating peripheral circulation, particularly in the distal parts of the body, such as the hands and feet. This impairment in the blood flow leads to thermoregulatory disorders in these areas (Holowatz et al., 2010). Furthermore, with age, the sweating threshold increases and the ability to evaporate sweat decreases (Millyard et al., 2020). These factors directly affect the body's ability to dissipate heat.

With regard to physical activity, studies on the body's heat dissipation capacity during exercise indicate that this mechanism begins to decline after the age of 40, with significant differences observed in the 45–49 and 50–55 age categories (Larose et al., 2013a). Age-related deterioration of performance-related variables, including thermoregulatory efficiency, has been associated with reduced exercise tolerance, slower recovery, and higher perceived exertion. In older adults, a decrease in maximal oxygen uptake (VO_2__max_), stroke volume, pulmonary diffusing capacity, as well as muscle mass and strength is observed. The decline in these variables reduces the body's ability to generate energy required during intense exercise (Vigorito and Giallauria, 2014). Impaired heat dissipation caused by a disrupted thermoregulatory mechanism contributes to a faster rise in core body temperature (Larose et al., 2013a, 2013b), which directly affects physical performance and, in the context of recovery, prolongs the return to homeostasis (Racinais and Oksa, 2010). Importantly, most of the evidence describing age-related impairments in thermoregulation is derived from untrained or recreationally active populations. The hypothesis of potential differences in thermal response among the various age categories of Master athletes is supported by research (Larose et al., 2014) according to which as a result of a reduced rate of heat dissipation predominantly during exercise, 40–70-yr age groups are storing between 60–85 and 13–38% more heat than the 20–30-yr age group despite the conditions. The reason is that older individuals have been shown to exhibit higher core temperatures under the same thermal load and to cool down more slowly due to reduced sweating and lower skin blood flow. Whether similar mechanisms operate to the same extent in highly trained older athletes, particularly under competitive conditions, remains insufficiently explored, which justifies analyzing thermal responses across Master age groups (Núñez-Rodríguez et al., 2025).

Research has shown that regular physical exercise has a positive effect on slowing down the adverse changes associated with aging (Kawamura et al., 2024). The benefits of regular sports training help maintain (or even improve) muscle mass and strength (Marzuca-Nassr et al., 2023), and counteract the decline in VO_2__max_ (Vigorito and Giallauria, 2014) which is a key indicator of cardiovascular performance. Regular sports activity may also slow down age-related changes in tendon tissues (Bravo-Sánchez et al., 2021) and limit the decline in thermoregulatory control in older adults (Balmain et al., 2018; Kenney, 2003). As a result, Master athletes represent a unique model of successful aging, in which the negative physiological consequences of aging are at least partly counterbalanced by long-term training adaptations (Tanaka and Seals, 2008).

Despite the proven benefits of regular physical activity in delaying the effects of aging, critical periods occur throughout human ontogenesis, characterized by significant hormonal changes that affect the functioning of various bodily systems. One such a period is around the age of 50, which involves the onset of menopause in women and andropause in men. Both periods are associated with a sharp decline in estrogen and testosterone levels, hormones that affect the thermoregulatory center in the hypothalamus (Singh et al., 2013). Another critical period occurs around the age of 70. Physical performance declines linearly until about the age of 70, after which the rate of decline accelerates (Baker et al., 2009; Reaburn, 2009). This period is also marked by a significant decrease in testosterone levels (Stanworth and Jones, 2008). As a result, changes occur in energy levels, strength, physical fitness and psychomotor function (Zirkin and Tenover, 2012). In the context of thermoregulatory dysfunction, it is important to note the reduced ability of the body to maintain water-electrolyte balance. This is due to impaired kidney function—by the age of 70, the kidneys filter approximately 50% less than they did at the age of 30 (Reaburn, 2009). These changes also contribute to decreased athletic performance (Lazarus and Harridge, 2017). It should be acknowledged, however, that studies involving athletes aged over 70 years are often limited by smaller sample sizes, which may reduce statistical power when examining age-related differences.

Therefore, the assessment of motor functions, muscle-tendon responses, and thermoregulatory adaptations becomes an essential component of health prevention, personalized training processes, and rehabilitation in older adults. At the same time, technological advancements allow for the use of sophisticated, non-invasive methods to monitor these physiological variables. Thermography appears to be a useful tool for assessing the body’s response in terms of changes in surface temperature, which reflect, among other things, the blood flow, muscle metabolism, and inflammatory processes (Galán Carracedo et al., 2019). In the context of Master athletics, thermographic assessment during high-intensity competitive exercise may provide insight into the current physiological state and potential dysfunctions that are not readily detectable under laboratory or training conditions, offering practical implications for training monitoring, recovery strategies, and load management (Adamczyk, 2023; Adamczyk et al., 2024).

Considering the described factors influencing changes in exercise capacity and performance related to alterations in thermoregulation, mainly resulting from the decreasing efficiency of sweat glands and the ability of cutaneous blood vessels to dilate, lower cardiac output and a decline in VO_2__max_, reduced muscle mass, and a weakened catecholaminergic response (Hassani et al., 2024), older runners respond to exercise with higher body temperatures and less effective cooling mechanisms compared to younger individuals, which may also result in functional asymmetries in this regard. These responses are typically studied in training contexts. However, competition requires maximal or near-maximal effort. Despite the growing participation of older athletes in competitive events, there remains limited evidence on how maximal competitive exercise affects surface temperature distribution in trained Master athletes and whether age-related differentiation persists under such conditions. Therefore, the primary aim of the present study was to characterize exercise-induced changes in lower-limb skin surface temperature in Master athletes. A secondary, exploratory objective was to describe potential age-related patterns in these thermal responses across different age categories.

## Methods

### Participants

Master track and field athletes are defined as 35 years of age and older. Athletes are divided in 5-year age groups starting at the age of 35 and the classification is as follows: 35–39, 40–44, 50–54, etc. In total 148 male Master track & field athletes participating in the European Masters Athletics Championships Indoor (Toruń 2024) were enrolled in the study, all of them with the BMI within the norm (18.5–24.9 kg/m^2^). Due to the intermittent nature of exertion in certain events, athletes competing in throwing and jumping events were excluded from the study, as the specific structure of these competitions involves repeated short bursts of effort with rest intervals of highly variable duration.

All participants were Caucasian. The mean age of the participants was 55.56 years (±15.07 years), and their average training experience was 18.08 years (±31.85 years). During the considered season, participants trained an average of 6.6 hours per week (±7 h). Athletes represented various track events, including sprints (60 m, 200 m, 400 m, 60 m hurdles) as well as middle- and long-distance events (800 m, 1500 m, 3000 m, cross-country).

Based on the study hypothesis and the rationale outlined in the introduction, participants were divided into three age groups:

- M35–45, n = 68 (Masters M35, M40 and M45 categories with athletes aged 35 to 49 years), mean age: 40.23 ± 3.93 years;
- M50–65, n = 64 (Masters M50, M55, M60 and M65 categories with athletes aged 50 to 64 years), mean age: 58.13 ± 6.29 years;
- M70+, n = 16 (Masters M70 and older categories with athletes over 70 years old), mean age: 74.06 ± 4.91 years.

In order to participate in the study, all subjects had to complete and sign an informed consent form in which they were informed about the benefits and potential risks of the study. In addition, they signed the authorization to use their data for academic and research purposes while maintaining their anonymity. The Code of Ethics of the World Medical Association (Declaration of Helsinki) for experiments involving humans was applied. The project was approved by the Bioethics Committee of the National Institute of Public Health, National Institute of Hygiene, Warsaw, Poland (protocol code: 1/2021; approval date: 18 January 2021).

### Design and Procedures

The data collection was carried out indoor, close to the warm-up zone of the facilities of the indoor track of Arena Toruń. In the room an ambient temperature of 20.1 ± 0.3°C was recorded. An ambient humidity of 48.0 ± 2.5% was registerd.

After arrival and collection of personal data, participants underwent a 10–15-min thermal adaptation period to acclimate to the ambient temperature, which is in line with the time required to stabilize thermographic images at rest (Marins et al., 2014). During this adaptation phase, athletes rested passively without wearing track clothing that would cover the surface of the lower limbs. Following acclimatization, two thermal images of each athlete’s lower limbs were taken—one from the anterior view and one from the posterior view. This imaging procedure was conducted prior to competition, and this condition was henceforth referred to as the *REST* state.

Subsequently, athletes completed an individualized warm-up following the RAMP (Raise, Activate, Mobilize, Potentiate) protocol. This protocol consists of four phases (Jeffreys, 2007): *Raise*: this part of the warm-up is focused on increasing values of key physiological variables, including the blood flow, muscle temperature, core temperature, muscle elasticity, and the quality of neuromuscular activation and conduction. This is achieved through intentional use of low-intensity movements and key locomotor patterns; *Activate*: this phase is targeted at activation of key muscle groups; *Mobilize*: it consists of mobilization of key joints and range of motion required for the upcoming activity; *Potentiate*: this part includes high-intensity exercises that are highly specific to the demands of the particular sport. Preparation according to the RAMP protocol is considered one of the most effective warm-up strategies (Vadher et al., 2024). All participants performed a warm-up in accordance with the RAMP structure, but we did not interfere with its length so that each athlete could prepare for the start in the best possible way for them individually.

Immediately after completing the warm-up, athletes participated in their main event at the Championship race. Given their participation in the European Masters Athletics Championships Indoor, it was reasonable to assume that the race was performed at maximum intensity by each athlete.

**Figure 1:**
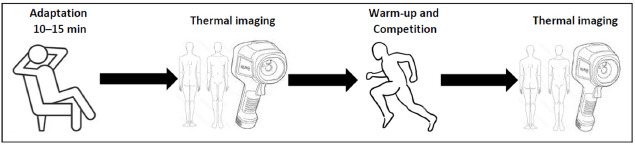
Study timeline with subsequent activities.

Directly after the race (approximately 5 min, although this time may have varied slightly depending on the participants due to different procedures for transitioning from the track to the laboratory), athletes moved to the measurement stand, where thermographic images of the lower limbs were taken again. The study protocol is presented in Figure 1.

**Figure 2:**
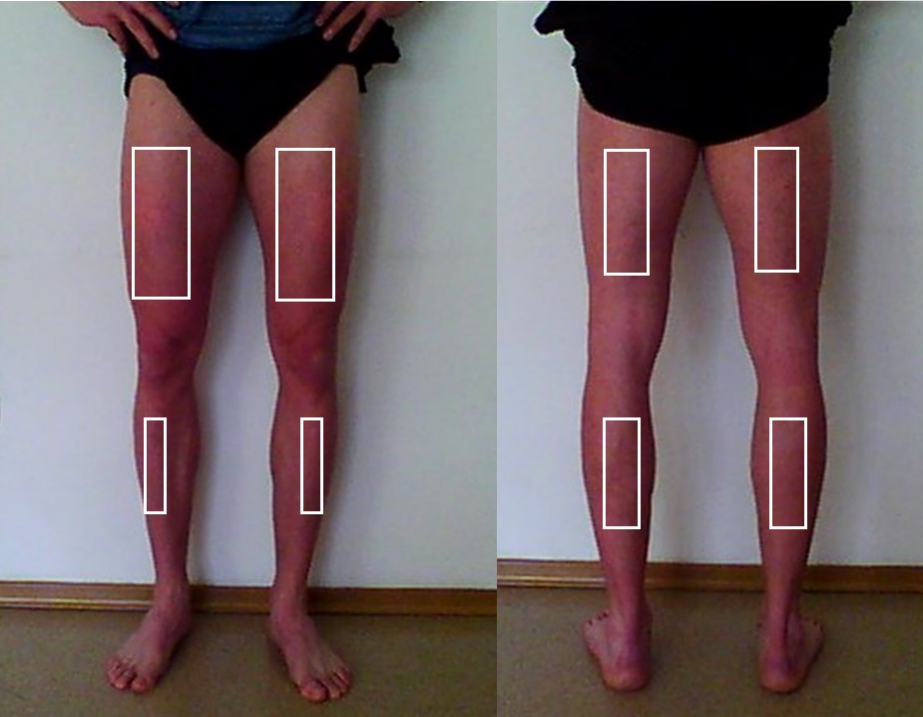
The anterior and the posterior view with marked regions of interest (ROI's) taken for analysis.

Infrared thermographic images of the anterior and posterior surfaces of the lower limbs were taken for each participant while in an upright standing position. The analysis focused on cutaneous temperature (T_sk_°C) within predefined regions of interest (ROIs) corresponding to the following muscle groups: tibialis anterior, gastrocnemius, biceps femoris, and rectus femoris (Figure 2).

**Figure 3:**
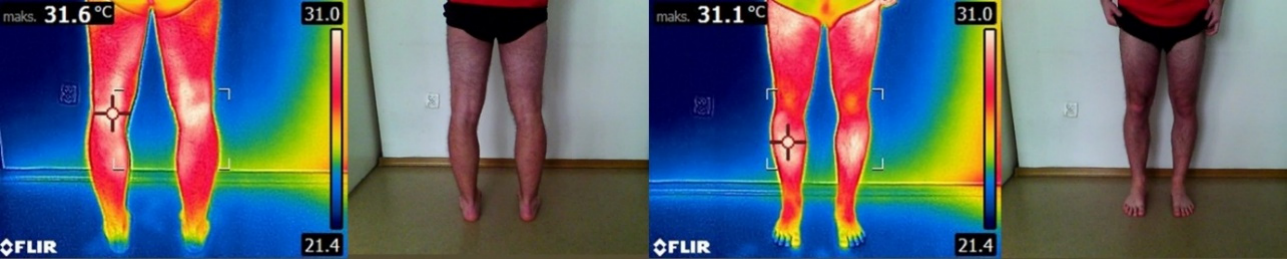
Example of thermal images taken in the anterior view.

To ensure consistency in thermographic assessment pre- and post-exertion, ROIs were delineated using physical markers, thereby standardizing the anatomical sites evaluated across all imaging sessions. The markings were made by a certified ISAK specialist, which increased the reliability of ROI placement. Throughout both the thermal adaptation phase and the period between physical exertion (race) and subsequent image acquisition, the target skin areas remained exposed to facilitate the accurate thermal pattern. For subsequent analysis, the mean skin temperature values of the marked ROIs were computed separately for the anterior and posterior sides of both the right and left lower limbs (Figure 3).

Thermal imaging was conducted using a FLIR E8 thermal camera (FLIR Systems, Sweden), following the 'Thermographic Imaging in Sports and Exercise Medicine (TISEM)' protocol outlined by Moreira et al. (2017). The device operates within a temperature range of −20 to +250°C, offering accuracy of ±2°C or ±2%, thermal sensitivity of less than 0.05°C, a 9-Hz refresh rate, and a 320 × 240 pixel Focal Plane Array resolution. The camera was positioned 2.5 m from the subject during image capture. Data analysis was carried out using FLIR Tools software.

### Statistical Analysis

Statistical analysis of the obtained results was conducted using STATISTICA 13 software (TIBCO Software Inc., 2017, USA). Descriptive statistics were used to determine mean values, standard deviations, and 95% confidence intervals for the analyzed variables. The Shapiro-Wilk test was applied to assess the normality of data distribution. Homogeneity of variance was evaluated using the Levene’s test.

Comparisons of mean skin temperature values before and after the race were performed using the Student’s *t*-test for dependent samples. When at least one variable did not meet the assumption of normality, the Wilcoxon signed-rank test was used. Differences in mean values among three groups within the same measurement were analyzed using one-way analysis of variance (ANOVA). In cases where the assumptions of normality or homogeneity of variance were violated in at least one group, the Kruskal-Wallis test was applied.

Effect sizes for the parametric *t*-test (both dependent and independent variables) were calculated using Cohen’s *d* with small effect (0.2), medium effect (0.5), and large effect (0.8). Meanwhile, for the Wilcoxon test, the coefficient of two-way serial correlation for matched pairs (r_c_) was applied, and for single factor ANOVA eta^2^, the Glass's rank biserial (r_g_) correlation coefficient was used. For both of these indicators, the following magnitude values were adopted: weak effect (0.1), moderate effect (0.3), and strong effect (0.5).

To determine the required sample size, an a priori sample size calculation was performed using G*Power (v. 3.1.9.7; Düsseldorf, Germany). Based on previous studies about the effect of effort on skin temperature, estimation for a difference between two dependent means, using a large effect size of *d* = 0.7, α error of 0.05, 1 – β = 0.8, with one group and two testing time points, a minimum sample size of n = 15 was determined. Additionally, considering the study design, one-way ANOVA was used to compare three groups within the same measurement. Assuming a large effect size (f = 0.25), the minimum required sample size was calculated as *n* = 84.

## Results

The warm-up and competition in the 35–45 age group caused a decrease in skin temperature (T_sk_) and significant changes were noted in T_sk_ in the area of the rectus femoris muscle in both the left (*p* = 0.013; *d* = 0.3) and right (*p* = 0.007; *d* = 0.3) lower limbs (Table 1).

**Table 1:** Thermal response to the warm-up and competition in the 35–45 age group.

Muscle group	M/SD CI ± 95%	t / Z	*p*	Cohen’s *d* /rg	Effect size
Tibialis anterior left at rest	31.03 ± 1.07 (30.76:31.28)	1.103	0.274	0.1	S
Tibialis anterior left after the race	30.78 ± 1.83 (30.32:31.23)				
Tibialis anterior right at rest #	31.08 ± 1.20 (30.78:31.37)	1.663	0.096	0.2	S
Tibialis anterior right after the race #	30.59 ± 1.96 (30.1:31.07)				
Rectus femoris left at rest	31.22 ± 1.08 (30.94:31.48)	2.569	0.013*	0.3	M
Rectus femoris left after the race	30.68 ± 1.68 (30.26:31.09)				
Rectus femoris right at rest	31.63 ± 1.37 (31.29:31.97)	2.807	0.007*	0.3	M
Rectus femoris right after the race	31.00 ± 1.72 (30.56:31.42)				
Gastrocnemius left at rest	31.11 ± 1.2 (30.81:31.4)	1.118	0.268	0.1	S
Gastrocnemius left after the race	30.63 ± 1.75 (30.19:31.06)				
Gastrocnemius right at rest	31.03 ± 1.3 (30.71:31.35)	2.385	0.020*	0.3	M
Gastrocnemius right after the race	30.48 ± 1.91 (30.01:30.95)				
Biceps femoris left at rest	31.06 ± 1.16 (30.77:31.35)	2.536	0.014*	0.3	M
Biceps femoris left after the race	30.55 ± 1.65 (30.14:30.95)				
Biceps femoris right at rest #	31.03 ± 1.63 (30.63:31.43)	0.188	0.851	0.0	S
Biceps femoris right after the race #	30.98 ± 1.61 (30.57:31.37)				

M: Mean T_sk_ [°C]; SD: standard deviation; CI: confidence interval; t/Z: the “t” parameter is a test statistic that determines the strength of the difference between the means in the compared groups test T.; the “Z” stands for standardized test statistic, which is used to assess the significance of differences between two groups in the Wilcoxon test; p: p-value, level of statistical significance: * p ≤ 0.05; effect size for student Test Cohen’s d or the Wilcoxon test: rg; S: small, M: medium, L: large; # abnormal distribution

**Figure 4:**
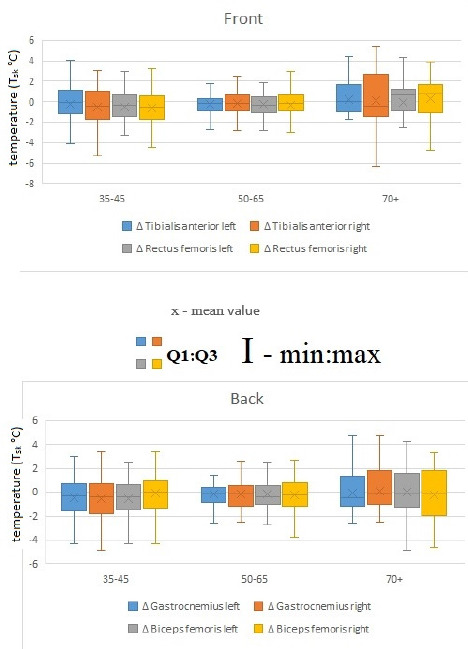
Changes in the thermal portrait (ΔT_sk_ [°C]) in response to exercise in the 35–45, 50–65, and 70+ age groups in the analyzed regions of interest in the front and the back view.

A significant decrease in skin temperature was also observed in the left biceps femoris (*p* = 0.014; *d* = 0.3), accompanied by a similar contralateral reaction in the area of the right gastrocnemius (*p* = 0.020; *d* = 0.3). Interestingly, the magnitude of the statistically significant changes observed for the left rectus femoris and the right gastrocnemius was identical (ΔT_sk_ = 0.54°C and 0.55°C, respectively), and very similar for the biceps femoris (ΔT_sk_ = 0.51°C). The greatest decrease in T_sk_ was recorded for the right rectus femoris (ΔT_sk_ = 0.63°C) (Figure 4).

Post-exercise measurements showed no significant variation in skin temperature for the 50–65 and 70+ age groups. In the 50–65 age group after exercise, lower skin surface temperature values were observed and (as in 35–45 age group) the most reactive area to physical effort related to the warm-up and competition was the right biceps femoris, although the temperature change (ΔT_sk_ = 0.32°C) (Figure 3) was not statistically significant. In both the 50–65 and 70+ age groups, none of the post-exercise temperature changes were statistically significant (Tables 2–3). However, symptomatically, the oldest athletes showed higher surface temperatures in five out of eight regions of interest (ROIs) after the competition compared to resting conditions. Exceptions included the rectus femoris, biceps femoris, and gastrocnemius areas, but only on the right lower limb (Table 3).

**Table 2:** Thermal response to the warm-up and competition in the 50–65 age group.

Muscle group	M/SD CI ± 95%	t / Z	*p*	Cohen’s *d* /rg	Effect size
Tibialis anterior left at rest	31.22 ± 1.16 (30.92:31.5)	1.717	0.086	0.2	S
Tibialis anterior left after the race	30.97 ± 1.38 (30.62:31.31)				
Tibialis anterior right at rest	31.38 ± 1.43 (31.02:31.73)	0.654	0.513	0.1	S
Tibialis anterior right after the race	31.17 ± 1.95 (30.68:31.65)				
Rectus femoris left at rest	31.15 ± 1.23 (30.84:31.45)	1.243	0.214	0.1	S
Rectus femoris left after the race	30.97 ± 1.48 (30.6:31.34)				
Rectus femoris right at rest #	31.32 ± 1.05 (31.05:31.58)	1.917	0.055	0.2	S
Rectus femoris right after the race #	31.05 ± 1.34 (30.71:31.38)				
Gastrocnemius left at rest	31.12 ± 1.23 (30.81:31.42)	1.022	0.307	0.1	S
Gastrocnemius left after the race	30.97 ± 1.36 (30.62:31.3)				
Gastrocnemius right at rest	31.02 ± 1.30 (30.69:31.33)	1.070	0.285	0.1	S
Gastrocnemius right after the race	30.90 ± 1.45 (30.53:31.26)				
Biceps femoris left at rest	31.08 ± 1.19 (30.78:31.37)	0.661	0.509	0.1	S
Biceps femoris left after the race	30.95 ± 1.33 (30.61:31.28)				
Biceps femoris right at rest #	31.65 ± 1.40 (31.29:31.99)	1.569	0.122	0.2	M
Biceps femoris right after the race #	31.33 ± 1.89 (30.85:31.8)				

M: Mean T_sk_ [°C]; SD: standard deviation; CI: confidence interval; t/Z: the “t” parameter is a test statistic that determines the strength of the difference between the means in the compared groups test T.; the “Z” stands for standardized test statistic, which is used to assess the significance of differences between two groups in the Wilcoxon test; p: p-value, level of statistical significance: * p ≤ 0.05; effect size for student Test Cohen’s d or the Wilcoxon test: rg; S: small, M: medium, L: large; # abnormal distribution

**Table 3:** Thermal response to the warm-up and competition in the 70+ age group.

Muscle group	M/SD CI ± 95%	t / Z	*p*	Cohen’s *d* /rg	Effect size
Tibialis anterior left at rest	31.21 ± 1.28 (30.53:31.89)	0.207	0.836	0.1	S
Tibialis anterior left after the race	31.46 ± 1.75 (30.53:32.39)				
Tibialis anterior right at rest	31.19 ± 1.23 (30.53:31.84)	0.078	0.938	0.0	S
Tibialis anterior right after the race	31.24 ± 1.67 (30.35:32.13)				
Rectus femoris left at rest	31.24 ± 1.55 (30.41:32.07)	0.026	0.979	0.0	S
Rectus femoris left after the race	31.36 ± 2.14 (30.22:32.5)				
Rectus femoris right at rest	31.22 ± 1.18 (30.59:31.84)	0.595	0.552	0.2	S
Rectus femoris right after the race	31.19 ± 2.11 (30.06:32.3)				
Gastrocnemius left at rest	31.24 ± 1.04 (30.68:31.79)	1.008	0.313	0.3	M
Gastrocnemius left after the race	31.58 ± 1.57 (30.74:32.4)				
Gastrocnemius right at rest	31.14 ± 1.36 (30.41:31.86)	0.414	0.679	0.1	S
Gastrocnemius right after the race	31.06 ± 1.96 (30.01:32.1)				
Biceps femoris left at rest	31.16 ± 1.39 (30.42:31.9)	0.103	0.918	0.0	S
Biceps femoris left after the race	31.29 ± 2.11 (30.16:32.41)				
Biceps femoris right at rest	31.28 ± 1.05 (30.72:31.83)	0.426	0.670	0.1	S
Biceps femoris right after the race	31.08 ± 1.74 (30.15:32.01)				

M: Mean T_sk_ [°C]; SD: standard deviation; CI: confidence interval; t/Z: the “t” parameter is a test statistic that determines the strength of the difference between the means in the compared groups test T.; the “Z” stands for standardized test statistic, which is used to assess the significance of differences between two groups in the Wilcoxon test; p: p-value, level of statistical significance: * p ≤ 0.05; effect size for student Test Cohen’s d or the Wilcoxon test: rg; S: small, M: medium, L: large; # abnormal distribution

The comparison of potential differences in the thermal profile between age groups revealed certain trends, particularly between the youngest group of athletes and the others. However, these trends appeared only after the completion of physical exertion. Statistical analysis did not indicate that these differences were significant (*p* > 0.05), either in the resting condition measurements or immediately after the run (Table 4).

**Table 4:** Variation in the thermal portrait between particular age groups, *p* values.

Age group	Time point	Tibialis ant. left	Tibialis ant. right	Rectus femoris left	Rectus femoris right	Gastro. left	Gastro. right	Biceps femoris left	Biceps femoris right
35–45 vs. 50-65	Before	0.165	0.166	0.284	0.478	0.160	0.161	0.469	0.101
	After	0.247	0.105	0.082	0.148	0.111	0.083	0.066	0.271
35–45 vs. 70+	Before	0.298	0.172	0.496	0.107	0.162	0.368	0.353	0.229
	After	0.089	0.101	0.188	0.103	0.217	0.089	0.075	0.415
50–65 vs. 70+	Before	0.495	0.412	0.376	0.102	0.480	0.169	0.371	0.379
	After	0.155	0.251	0.403	0.298	0.433	0.247	0.262	0.430

The assessment of asymmetry by comparing the mean temperatures in the analyzed locations showed no significant differences (*p* > 0.05) between the right and left lower limbs, regardless of the group or the measurement time point. As symptomatic, by far the smallest result dispersion (SD) occurred in the 70+ age group, although an exception might be the right tibialis anterior, especially in the post-race measurement.

## Discussion

The aim of this study was to analyze the thermal response to competitive effort in Master athletes of different age groups, with particular emphasis on potential differences between them. In light of the available literature, it is known that the aging process is associated with a progressive deterioration of physiological functions, including thermoregulatory mechanisms, which begin to deteriorate after the age of 40 (Larose et al., 2013a, 2013b). At the same time, much evidence suggests that regular physical exercise can effectively counteract age-related changes (Kawamura et al., 2024; Tanaka and Seals, 2008). In this context, Master athletes are increasingly seen as models of the so-called successful aging (Geard et al., 2017, 2021). A recent study has shown that in trained Master athletes, maximal exercise does not cause significant disturbances in thermoregulation or increased muscle stiffness, suggesting the preservation of adaptive properties of the musculoskeletal system despite advanced age (Adamczyk et al., 2024).

Our study results showed that the most noticeable changes in skin surface temperature (T_sk_) after exercise occurred in the 35–45 age group. Statistically significant decreases in T_sk_ were recorded over the rectus femoris muscle in both the left (ΔT_sk_ = 0.54°C) and right lower limbs (ΔT_sk_ = 0.63°C). Additionally, changes were observed in the area of the left biceps femoris muscle (ΔT_sk_ = 0.51°C) and the contralateral (right) gastrocnemius muscle (ΔT_sk_ = 0.55°C), indicating the presence of cross-reactions in the thermal response. The observed decrease in skin surface temperature (T_sk_) in the 35–45 age group following the race likely reflects an exercise-induced redistribution of the blood flow, where active muscles receive priority perfusion and cutaneous perfusion is transiently reduced, leading to a drop in T_sk_ immediately post-effort. During intense exercise, the skin blood flow decreases due to vasoconstriction in favor of muscle circulation, which can manifest as lower surface temperatures in areas overlying active muscle groups (Fernández-Cuevas et al., 2023). Such results may also stem from the specifics of indoor track running, where the presence of banked turns leads to asymmetrical loading of the lower limbs (Adamczyk et al., 2024). Pietraszewski et al. (2021) concluded that sprinting on curves placed greater demands on the inner lower limb than the outer one, although it should be noted that the degree of these demands depended on the curve radius. In those studies, in contrast to the results of the present study, the most heavily loaded muscle group was the gastrocnemius of the left leg, i.e., the inner leg.

In the 50–65 and 70+ age groups, no statistically significant changes in surface temperature after exercise were recorded. Nevertheless, in the oldest participants, a tendency toward increased surface temperature was observed in five of the eight analyzed regions, which should be interpreted as a speculative observation rather than a confirmed effect. This pattern may suggest—but does not conclusively demonstrate—a reduced efficiency of heat dissipation mechanisms and a slower return to thermal equilibrium. Such indications align with previous scientific reports pointing to the deterioration of thermoregulatory capacity with age. This is caused, among other factors, by a reduced thermal response such as the sweating mechanism or prolonged heat dissipation time (Balmain et al., 2018; Kenney et al., 2021). Older individuals also show impaired skin blood flow, which means a slower skin response to heat stress (Petrofsky et al., 2009). Overall, these observations point to a potential reduction in thermal capacity and reactivity, but should be considered speculative given the lack of statistical significance.

Comparative analysis between age groups did not reveal statistically significant differences, although it is worth noting that temperature decreases appeared larger in the 35–45 age group, a trend that should be interpreted cautiously. This may be related to the potentially greater thermal capacity of this age group of athletes. Supporting this thesis are the findings of Adamczyk et al. (2016), where athletes of higher sports levels showed a similar pattern of lowering of the body temperature. It should be added that physically active individuals generally display better heat dissipation mechanisms than non-athletes (Kapoor et al., 2023). Another study supporting this relationship demonstrated that a higher level of aerobic endurance correlated with greater heat loss (Lamarche et al., 2018). Thus, while these results are consistent with the idea that an effective ability to dissipate excess heat is associated with an efficient response to thermal stress, these interpretations remain speculative and should be treated as such (Adamczyk et al., 2016). In summary, athletes in the 35–45 age group exhibit a stronger thermal response, which may indicate a properly functioning thermoregulatory mechanism and greater physical fitness. On the other hand, reduced thermal reactivity in older athletes provides evidence of progressive limitations of this mechanism due to the aging process. Based on these findings, in trained athletes, temperature decreases more rapidly, which may be interpreted as a sign of greater thermal capacity—that is, the body’s ability to effectively cope with excess heat during and after exercise (Reilly et al., 2006).

The results of the study provide evidence that long-term sports training effectively mitigates the negative effects of aging on thermoregulatory functions. The most important phenomenon observed was a relatively small number of statistically significant differences in thermal response among age groups (35–45, 50–65, and 70+ years), which is in contrast to population data and suggests a protective effect of regular training (Best et al., 2012). Statistical analysis showed no significant differences in skin temperature among groups both at rest and after exercise, which indicates preserved thermal homeostasis regardless of age. Of course age-related impairments in thermoregulatory function are well-documented, with primary aging associated with reduced sweat rates and cardiovascular adjustments that can compromise heat loss and increase heat-related risk during exercise. However, high aerobic fitness enhances body temperature regulation via improved sweating and cardiovascular responses (Seo et al., 2024), meaning that older athletes with maintained fitness are better suited for heat stress than their less fit peers, even if some age-related declines persist (Kenney et al., 2021). This phenomenon can be interpreted as the effect of physiological adaptation induced by systematic physical activity, including, among others, maintaining cardiovascular fitness (Bahls et al., 2012; Vigorito and Giallauria, 2014), maintaining efficient peripheral circulation and elasticity of blood vessels (Shibata et al., 2018), increasing the activity and density of sweat glands and improving their response to an increase in body temperature, which allows faster and more effective heat dissipation through sweat evaporation (Gagnon and Kenny, 2012), increased blood flow through the skin, which allows for more efficient heat dissipation (Shibasaki and Crandall, 2010) and the level of hydration as regular physical activity promotes better hydration (Vila et al., 2024). It is also worth emphasizing the importance of metabolic and hormonal adaptations. Regular exercise can influence the regulation of heat stress hormones (e.g., aldosterone, vasopressin), which control water and electrolyte balance, and thus the efficiency of sweating and the maintenance of circulating blood volume (Sawka et al., 2011).

It is worth noting that only the youngest group of subjects showed a significant decrease in skin temperature after exercise, especially in the rectus femoris, biceps femoris and gastrocnemius muscles, which can be interpreted as a model thermoregulatory response resulting from active redistribution of the blood flow (Charkoudian, 2003). The lack of such changes in the older groups does not necessarily indicate dysfunction, but reflects the economical work of the circulatory system and the stability of thermoregulatory mechanisms. This may also be evidenced by the lack of differences in resting temperature among the groups, as well as the lack of extreme fluctuations in skin temperature after exercise (Best et al., 2012; Lee et al., 2024). Additionally, the results may have been influenced by conducting measurements under competition performance conditions, which differ from controlled settings in a research facility. The maximal effort associated with competition likely provides a more realistic representation of the body’s physiological responses than even the most carefully simulated laboratory conditions.

Literature indicates that with age, increasing thermal asymmetries are observed in the average population, especially in the lower limbs, which is associated with deterioration of microcirculation (Inoue and Shibasaki, 1996). These disorders lead to local differences in tissue perfusion and thus, discrepancies in skin surface temperature.

In this study, no statistically significant differences in skin temperature between the limbs were found, regardless of the group affiliation, the measurement location or time of measurement. This result, by showing thermal symmetry in each group of Masters studied, might indicate the preventive effect of sports training (Yue et al., 2025).

The proposed mechanisms explaining preserved thermoregulation in Master athletes are plausible, but should be interpreted with caution given the cross-sectional design of the study. The preserved thermoregulatory variables observed in older athletes may support and are broadly consistent with previous research, which consistently highlights that Master athletes maintain a notably higher level of physical fitness, superior overall health, and enhanced social engagement compared to their age-matched peers who lead more sedentary lifestyles. This elevated physical condition likely contributes to the efficient functioning of physiological systems involved in temperature regulation, including cardiovascular stability, sweat gland responsiveness, and skin blood flow adaptations. Together, these mechanisms may enable older athletes to cope more effectively with thermal stress, although causal relationships cannot be confirmed (Geard et al., 2021).

From a comparative perspective, the results of this study contradict numerous reports from the general population, in which, with age, one may observe, among others, an increased sweat threshold (Millyard et al., 2020), decreased heat dissipation efficiency and decreased skin circulation (Holowatz et al., 2010). The presence of these adverse phenomena was not confirmed in the group of Master athletes, which suggests that physical exercise plays a compensatory role in the natural aging processes. The average sports experience of the subjects of over 18 years is an additional argument for the long-term nature of this adaptation, which may include, among others, the preservation of capillary density, the efficiency of thermoregulatory receptors or the efficiency of sweat mechanisms (Geard et al., 2017).

### Limitations and Recommendations for Future Studies

Some limitations should be noted. We analyzed the thermograms manually but its performance by an experienced researcher minimized measurement error. In future studies it is worth considering automation using software (e.g., TermoHuman) which should speed up the analysis and minimize the risk of ROI reading error. Master athletes constitute a specific, highly motivated population with high training commitment, which may limit the generalizability of the results, and it is advised to rather apply them only to a well-trained male population. Future studies should therefore include comparisons with recreationally active groups (also female athletes) considering different environmental conditions (e.g., heat stress) to analyze other aspects of aging, such as cognitive and mental functions. Such studies could include other physiological variables such as core temperature measurements or internal load indicators.

One of the limitations is the difficulty in replicating the research; however, it should be taken into account that the measurements were carried out under real competition conditions rather than in a laboratory. It should be acknowledged as a limitation that thermographic images were acquired approximately 5 min after the race completion, as the timing of post-exercise skin temperature assessment is known to influence the measured thermal response and may introduce variability among subjects or when compared to protocols with immediate imaging (Vieira et al., 2020). Therefore, although the conditions were similar for all participants, slight differences in the protocol timeline may have occurred. Additionally, training performed by athletes was not analyzed, which could have enriched the analysis. However, in the context of the conducted study, the content of training—and particularly the distance—appears to be of secondary importance, serving only as a means of providing the stimulus. It is the frequency and repetition of exercise, rather than its type, that determine the level of heat acclimation (Périard et al., 2015). Finally, the study is marked by a significantly smaller number of the oldest competitors (70+) which might be the major limitation affecting between-group comparisons. Nevertheless, considering the official competition data (https://emaci2024.domtel-sport.pl/), their proportion in the study was actually greater than in the overall list of participants. Although an a priori power analysis was performed, the study may have been underpowered to detect between-group differences in the oldest age group, and thus, the results for this group should be interpreted with caution.

## Conclusions

In summary, the results of this study support the hypothesis that regular and long-term sports training effectively mitigates the negative effects of aging on thermoregulatory functions. The lack of significant differences among the age groups of Master athletes in response to maximal competitive effort provides evidence that physical exercise may be one of the most effective tools supporting successful aging. These observations fit into the broader concept of multidimensional aging, according to which maintaining physical and social functionality is also possible in elderly subjects, as long as it is accompanied by long-term involvement in sports and exercise.

In practical terms, thermography could be integrated into athlete monitoring through regular, serial screenings aimed at identifying atypical bilateral asymmetries or unusual patterns of local warming or cooling. Such thermal deviations may indicate early signs of overloading, inflammation, or impaired recovery, allowing for timely adjustments in training loads or preventive interventions. This application highlights thermography as a targeted tool for injury risk management rather than a general indicator of healthy aging (Côrte et al., 2019).

Our study is one of the first comprehensive analyses of the thermal response to physical exertion in Master athletes in track and field, taking age categories into account. The only significant changes in thermal response were observed in the 35–45 age group, which, in light of previous findings, may reflect the greater thermal capacity and faster adaptive responses typically seen in younger athletes during competitive exertion. On the other hand, a general, yet statistically non-significant, increase in temperature was observed in the oldest group alongside an overall thermal response more often associated with a decrease in temperature due to vasoconstriction of subcutaneous vessels and redistribution of the blood flow toward working muscles. The lack of significant differences in the thermal profile among the age groups should be interpreted cautiously and appears specific to this cohort of well-trained Master athletes. These observations may reflect the maintenance of sufficient physical fitness to cope with metabolic and thermal stress in this population. Therefore, for Master athletes, neither the post-50 nor the post-70 age period was associated with substantial changes in the thermal profile, suggesting that long-term training adaptations can support effective thermoregulation in competitive settings. These findings are particularly relevant for the design of training and recovery strategies in older athletes, highlighting that structured athletic activity may help mitigate age-related decrements in thermal responses following high-intensity exercise.
